# The Reliability and Agreement of the Fibromyalgia Survey Questionnaire in an Italian Sample of Obese Patients

**DOI:** 10.3389/fpsyg.2021.623183

**Published:** 2021-02-09

**Authors:** Giorgia Varallo, Ada Ghiggia, Marco Arreghini, Paolo Capodaglio, Gian Mauro Manzoni, Emanuele Maria Giusti, Lorys Castelli, Gianluca Castelnuovo

**Affiliations:** ^1^Psychology Research Laboratory, Istituto di Ricovero e Cura a Carattere Scientifico (IRCCS) Istituto Auxologico Italiano, Ospedale San Giuseppe, Piancavallo, Italy; ^2^Department of Psychology, Catholic University of Milan, Milan, Italy; ^3^Department of Psychology, University of Turin, Turin, Italy; ^4^Research Laboratory in Biomechanics and Rehabilitation, Orthopedic Rehabilitation Unit, Istituto di Ricovero e Cura a Carattere Scientifico (IRCCS) Istituto Auxologico Italiano, Ospedale San Giuseppe, Piancavallo, Italy; ^5^Faculty of Psychology, eCampus, University, Novedrate, Italy

**Keywords:** fibromyalgia (FM), obesity, fibromyalgia survey questionnaire, reliabiity, chronic pain, assessment

## Abstract

The Fibromyalgia Survey Questionnaire (FSQ) was self-administered by a sample of 207 Italian individuals with obesity to screen for fibromyalgia (FM). We aimed to investigate the inter-rater reliability and the agreement in the detection of FM symptomatology between the self-administered FSQ and the clinical interview conducted by a rheumatologist. All the patients were divided randomly into two groups (group A and group B): a rheumatologist first interviewed patients of group A and after 48 h, the patients completed the self-report FSQ. Patients of group B first completed the FSQ and 48 h later were interviewed by a rheumatologist. The agreement between the measurements was good with the Bland-Altman analysis showing low bias scores for the two subscales of the FSQ. Results showed that 33% of the sample satisfied the criteria for a diagnosis of fibromyalgia. The FSQ is a self-reporting measure that showed substantial reliability providing fast screening for FM symptomatology.

## Introduction

Fibromyalgia (FM) is a syndrome with unclear etiopathogenesis, characterized by chronic widespread pain, fatigue, sleep disturbances, and many other symptoms that significantly worsen quality of life (Bazzichi et al., 2020). FM affects 2.31% of the European population, and 2.22% of the Italian population (De Angelis et al., 2007; Cabo-Meseguer et al., 2017).

Several factors contribute to the intensity of its symptoms and to the resulting disability (Okifuji and Turk, 2002; Wallace, 2006), among swhich, weight is one of the most significant (McKendall and Haier, 1983; Khaodhiar et al., 2004). Evidence has shown that FM is closely associated with overweight and obesity (Ursini et al., 2011). According to several studies, 62–73% of patients with FM are overweight or obese (Yunus et al., 2002; Neumann et al., 2008; Okifuji and Hare, 2015). Specifically, a higher body mass index (BMI) seems to be positively correlated with disability (Yunus et al., 2002; Creed, 2020), physical dysfunction, tender point count, pain sensitivity, and sleep disturbances; but it is also negatively correlated with quality of life, tenderness threshold, physical strength, and flexibility (Neumann et al., 2008; Okifuji et al., 2010). However, research on individuals affected both by obesity and FM is still in its infancy. Early and precise diagnosis of FM is still difficult to obtain, due to the complex poly-symptomatology, and the different combination and severity of symptoms (Lawrence et al., 1998; Salaffi et al., 2016). Several classification and diagnostic criteria have been proposed, from the 1990 American College of Rheumatology (ACR) classification criteria to the 2010 ACR criteria, which consisted of three benchmarks:

*Criterion 1*: Widespread Pain Index (WPI) ≥ 7 and Symptom Severity Score (SSS) ≥5 or WPI 3–6 and SSS ≥9;

*Criterion 2*: Symptoms have been present at a similar level for at least 3 months;

*Criterion 3*: The patient does not have a disorder that would otherwise explain the pain.

The WPI includes 19 non-articular pain sites; meanwhile, the SSS measures the severity of three major symptoms (fatigue, trouble thinking or remembering, waking unrefreshed), and the severity of the somatic symptoms in general, rated by the physician. While for this classification, the SSS score required physician evaluation, a further change in 2011 removed the physician assessment of the extent of somatic symptoms and replaced it by a summary score of three self-reported symptoms, making it easier to use. This modification enabled researchers to use these criteria in epidemiological and clinical studies without the requirement for an examiner.

Lastly, after the 2016 revision, fibromyalgia may be diagnosed when all of the following criteria are met: (1) WPI ≥ 7/19 pain sites and SSS ≥ 5/12 or WPI between > 3–6/19 and SSS > 9/12; (2) symptoms have been present at a similar level for at least 3 months; (3) the patient does not have another disorder that would otherwise sufficiently explain the pain; and (4) generalized pain, defined as pain in at least four of five regions, is present (Wolfe et al., 2016).

Beyond the set of criteria used, the diagnosis of FM remains difficult; it requires differential diagnoses with several medical conditions, and the diagnostic delay can have a significant impact on the quality of life and emotional state of patients, as well as on health care and social costs (Di Franco et al., 2011). One of the most used tools for the evaluation of fibromyalgia symptoms is the Fibromyalgia Impact Questionnaire-Revised, FIQR (Bennett et al., 2009), which accurately measures the level of disability associated with the disease, especially in terms of function, global impact, and symptoms. This questionnaire is not suitable for the screening of fibromyalgia symptoms, according to the 2010/2011 ACR criteria.

Recently, the Fibromyalgia Screening Questionnaire (FSQ) has been proposed for this purpose to assess ACR conditions 1 and 2 on the two subscales: the WPI and the SSS (Häuser et al., 2012).

Our research question focused on evaluating whether the ability of the FSQ to detect fibromyalgia was equal to that of a clinical interview conducted by a rheumatologist. The clinical interview is based on the three criteria from 2010 reported above, for which the presence of an examiner is required; whereas the FSQ is based on the 2011 modification of the ACR criteria, assessing criteria 1 and 2, through the WPI and SSS scales (Häuser et al., 2012), and excluding criterion three, which can only be evaluated by a rheumatologist.

The objective of our study was to calculate the inter-rater reliability and the agreement between the Italian translation of the self-administered FSQ and the clinical interview conducted by a rheumatologist (see Wolfe et al., 2010), in detecting the symptomatology of FM in a sample of Italian patients with obesity and generalized pain.

## Materials and Methods

### Participants and Procedures

Participants of the study were recruited from May 2014 to September 2014 at the Istituto Auxologico Italiano –Istituto di Ricovero e Cura a Carattere Scientifico (Piancavallo–Italy), a clinic specializing in the rehabilitation of obesity and physical therapy. Consecutive patients were asked to take part in the study and give their informed written consent to the study, which had been approved by the Institutional Ethics Committee.

Demographic data (age, gender, educational level) were assessed using a self-report questionnaire. Two-hundred and twenty-five obese patients were recruited: 12 did not give their consent to the study and six patients were excluded from the analyses because of a high number of missing items; the final sample consisted of 207 subjects.

### Materials

The FSQ had been forward and back translated to ensure the semantic equivalence of the Italian and the English version by four rheumatologists, fluent in English, and experts in fibromyalgia. The clinical interview was conducted according to the 2010 ACR criteria (Wolfe et al., 2010). All the patients were divided randomly into two groups: a rheumatologist first interviewed group A and after 48 h, the patients completed the FSQ. Patients of group B first completed the FSQ and 48 h later were interviewed by a rheumatologist.

The questionnaire is divided in two subscales: the WPI is obtained by summing the body areas (out of 19 possible sites) in which the patient indicates they experienced pain during the preceding week, i.e., it can range from 0 to 19. The SSS consists of two parts:

*Part 1*: the patient grades the severity over the last week of three somatic symptoms (waking unrefreshed, disturbed cognition, and fatigue) on a scale of 0–3, resulting in a maximum score of 9;

*Part 2*: the patient grades the severity of the following three symptoms on a 4-point scale (0 = absent, 1 = slight, 2 = moderate, or 3 = severe), occurring during the previous 6 months: headaches, pain, or cramps in the lower abdomen and depression (a 0–12 total score).

Furthermore, a poly-symptomatic distress (PSD) scale can be also calculated by the sum of the two component scores, WPI and SSS (Wolfe et al., 2016).

### Statistical Analysis

All statistical analyses were performed using the Statistical Package for Social Science for Mac (SPSS-24, IBM SPSS Statistics for Macintosh, IBM Corp., Armonk, NY, USA).

Descriptive statistics were used to describe the socio-demographic and clinical data. Internal consistency of the total scales and relative subscales was assessed by Cronbach's α; values of 0.7 and higher were considered desirable (Nunnally and Bernstein, 1994).

Cohen's kappa statistic and intraclass correlation coefficient (ICC) were used to measure agreement between the two different tools, using a two-way mixed-effect model based on single ratings, for criteria 1 and 2. Mean estimations along with 95% confidence intervals (CI) were reported for each ICC. Furthermore, to measure continuous variable agreement (PSD, WPI, and SSS), we used Bland-Altman analysis (Bland and Altman, 1986): a scatter plot was constructed in which the differences between questionnaire and interview measurements were plotted on the y-axis and the average of the measures of two approaches on the x-axis. The mean difference in values obtained with the two approaches is the bias, represented by a central horizontal line on the scatter plot. Above and below the horizontal line, the 95% limits of agreement (LOA), expressed as the mean difference ±1.96, are represented: the smaller the range between these two limits, the better the agreement. The differences between the values were tested with a Student's *t*-test. A level of significance of *p* < 0.05 was considered.

## Results

Most participants were female (165 vs. 42 males; mean age: 63.2 ± 12.4). All participants had a diagnosis of obesity, with a level of BMI ≥ 30 (mean ± SD: 40.54 ± 6.45). More than half the patients (59%) had achieved a basic education and only 20 subjects (10%) had a tertiary education level.

Cronbach's alpha of the SSS was 0.710. Cohen's κ was run to determine if there was an agreement between the FSQ and the clinical interview conducted by a rheumatologist, in satisfying the criteria 1 and 2 for FM (see the above mentioned 2011 ACR criteria). Sixty-nine (33%) subjects were found positive by both the FSQ and interview vs. 103 patients that did not satisfy the criteria. There was good agreement between the two measurements, κ = 0.653 (95% CI, 0.55–0.757), with a *p* < 0.0005, index of a substantial strength of agreement (Altman, 1999). A minor discordant result was found for 16 patients, who were positive for the interview but not for the FSQ measurement.

A high degree of reliability on criterion 1 was also underlined by the interclass correlation. For the PSD scale, the average ICC measure was 0.899 with a 95% CI from 0.867 to 0.923, indicating good reliability (Koo and Li, 2016). Similarly, the average ICC measure for WPI was 0.888 (0.853–0.915) and for SSS was 0.851 (95% CI, 0.804 to 0.887).

**Figure 1:**
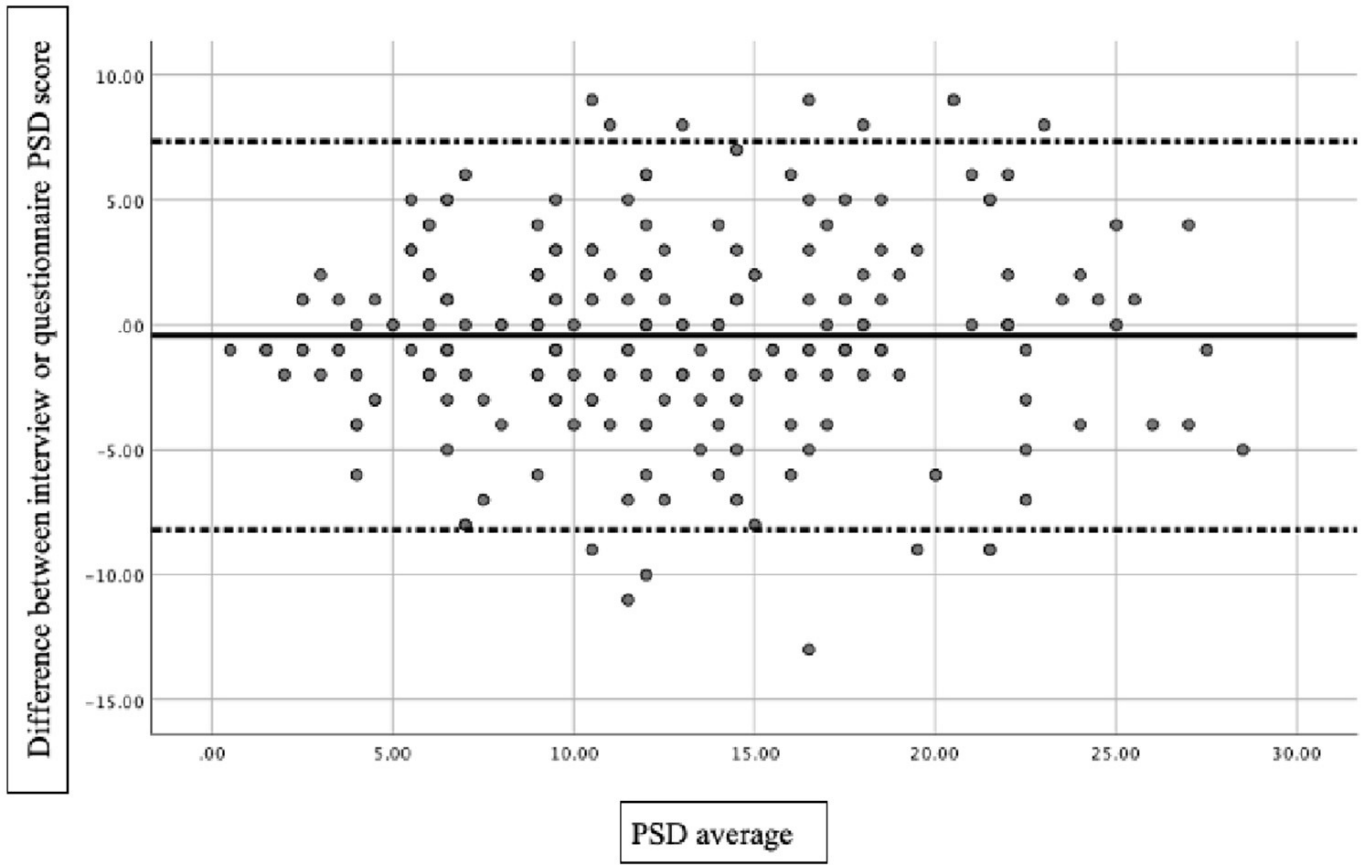
Bland-Altman 95% limits of agreement plot for PSD.

A low bias score between the two assessments was found: regarding PSD, a bias of 0.43 (*p* = 0.118) was found with the Bland-Altman 95% limits of agreement of −0.97 and 0.11 (Figure 1). Specifically, PSD detected by the FSQ was 12.5 ± 6.61, whereas the mean score at the interview was 13 ± 6.5. WPI scores were 7.41 ± 4.22 by the interview, slightly greater than by patient measures (7.26 ± 4.23) with a difference of 0.134 (*p* = 0.472). Similar values were found for the SSS, with a bias of 0.030 (*p* = 0.062): mean scores found by the FSQ were 5.29 ± 3.22 vs. 5.59 ± 4.22 by the interview.

## Discussion

The aim of this study was to evaluate the agreement and inter-rater reliability between the self-administered FSQ and a clinical interview conducted by a rheumatologist, for the detection of fibromyalgia symptoms. Since the results suggest that there is a good agreement and good inter-rater reliability between the two instruments, we can recommend the use of FSQ as a valid screening tool.

To date, there is no consensus on an ideal measurement to assess the severity of symptoms in FM or to monitor patients for symptoms, outcomes, or changes. Although criticized because it does not fully address all the aspects of FM, FIQR is a specific questionnaire for FM and addresses several and specified areas for this condition (Rivera and González, 2004; Bennett et al., 2009); however, it may not be sufficiently sensitive to changes.

FM diagnosis can be complex and challenging, and usually, its diagnosis requires a lot of time. Most patients are diagnosed on average 2.3 years after first presenting FM complaints to a physician (Marcus et al., 2013). The requirement for a physician's examination is a major limitation in understanding FM prevalence and characteristics. Therefore, the FSQ may be a useful tool to assist the physician's diagnosis and to accelerate the diagnostic process.

Indeed, a screening tool is necessary for use in settings where the presence of an interviewer would be difficult and can provide meaningful information on the overall status of FM symptomatology (Shir et al., 2012). Our results, similar to those of Wolfe et al. (2016), underlined that the FSQ appears to be an appropriate tool for the screening purposes of symptomatology.

While the results of the questionnaire and the interview appear to be comparable to the scores on the SSS and WPI scales, subtle differences were found in the satisfaction with the diagnosis. We must, however, remember that the clinical interview is based on the three criteria from 2010, in which the presence of an examiner is required; whereas the FSQ is based on the 2011 modification of the ACR criteria, assessing only criteria 1 and 2.

Patients affected by FM often undergo unnecessary medical examinations before their diagnosis is finally confirmed, and ineffective and incongruous treatments may be administered, due to delay and misdiagnosis.

We found an overall good agreement: specifically, for the PSD and the SSS scores, we found similar values of bias, while a significantly lower bias was found for the WPI scores, compared to Wolfe's (2016) results. Also, concerning the composition of our sample, we found that approximately a third of the patients satisfied the criteria for FM, in line with a previous study conducted on obese patients that reported a prevalence rate of 27.7% (Arreghini et al., 2014). Individuals affected by fibromyalgia and/or obesity may lie on the same syndrome continuum: it is known that a high body mass represents a risk factor for the development of chronic widespread pain (Creed, 2020); therefore, a vicious circle in the behavior of those suffering from obesity and fibromyalgia could be hypothesized.

Given the poor generalizability of our work, performed on a relatively small sample of patients with obesity, further validation studies in the Italian language and on a larger patient population are needed.

In conclusion, a self-reporting measure like the FSQ could provide a time-efficient method for the initial identification of patients with high pain sensitivity. Early diagnosis allows for the initiation of non-pharmacological approaches, such as psychotherapy or physical treatment, and could also reduce national healthcare costs.

## Data Availability Statement

The raw data supporting the conclusions of this article will be made available by the authors, without undue reservation.

## Ethics Statement

The studies involving human participants were reviewed and approved by Istituto Auxologico Italiano-IRCCS. The patients/participants provided their written informed consent to participate in this study.

## Author Contributions

GV and AG conceived and designed the study protocol with input from PC, MA, LC, and GC. GM carried out literature searches. AG designed and carried out the statistical analysis. GV, AG, MA, and EG interpreted the data and drafted the manuscript. EG and LC supervised the writing of the manuscript. All authors critically reviewed and contribute to the final version of the paper.

## Conflict of Interest

The authors declare that the research was conducted in the absence of any commercial or financial relationships that could be construed as a potential conflict of interest.
