# Supplementary material for: The Reliability and Agreement of the Fibromyalgia Survey Questionnaire in an Italian Sample of Obese Patients
Source: Front Psychol. 2021 Feb 9;12:623183. doi: 10.3389/fpsyg.2021.623183 (PMC7900136; doi:10.3389/fpsyg.2021.623183)
Supplement: Supplementary file 1 [file Table_1.DOCX]

**SUPPLEMENTAL MATERIALS**

**QUESTIONARIO FIBROMIALGIA**

1. Per favore indichi sotto se ha avuto dolori o indolenzimento durante gli ultimi 7 giorni in ognuna delle zone corporee indicate di seguito. Faccia attenzione a considerare la parte destra e sinistra separatamente.

| - Spalla sinistra | - Anca sinistra | - Mascella sinistra |
| --- | --- | --- |
| - Spalla destra | - Anca destra | - Mascella destra |
| - Braccio sinistro | - Coscia sinistra | - Torace |
| - Braccio destro | - Coscia destra | - Addome |
| - Avambraccio sinistro | - Gamba sinistra | - Area dorsale |
| - Avambraccio destro | - Gamba destra | - Area lombare |
|  |  | - Collo |

Totale *WPI/Widespread Pain Index* (0-19)

1. Per ognuno dei 3 sintomi elencati sotto, per favore indichi se e quanto ognuno di essi le ha dato problemi nel corso dell’ultima settimana.

| Stanchezza | 0 | 1 | 2 | 3 |
| --- | --- | --- | --- | --- |
| Sonno non ristoratore | 0 | 1 | 2 | 3 |
| Difficoltà di concentrazione e/o calo di memoria | 0 | 1 | 2 | 3 |

0 = nessun problema

1 = problemi lievi o moderati, generalmente moderati o intermittenti

2 = problemi moderati, considerevoli, spesso presenti e/o a moderata intensità

3 = problemi severi, penetranti, continui, che compromettono le normali attività della vita quotidiana

1. Durante gli scorsi mesi ha avuto qualcuno dei seguenti sintomi?

Dolori o crampi nella parte bassa addominale SI NO

Depressione SI NO

Mal di testa SI NO

Totale *SS/Symptom severity*: somma della gravità dei tre sintomi al quesito 2 più somma della severità dei sintomi somatici in generale (0-12)

1. Tutti i sintomi riferiti sono stati presenti con la stessa intensità per almeno 3 mesi?

- SI
- NO
